# Ketogenic diets in chronic kidney disease patients: a review for skeptics by skeptics

**DOI:** 10.1007/s40620-025-02285-7

**Published:** 2025-04-30

**Authors:** Claudia D’Alessandro, Domenico Giannese, Giorgina Barbara Piccoli, Vincenzo Panichi, Adamasco Cupisti

**Affiliations:** 1 Department of Clinical and Experimental Medicine, University of Pisa, Pisa, Italy; 2 Centre Hospitalier Le Mans, Le Mans, France

**Keywords:** Ketogenic diets, Chronic kidney disease, Obesity, Polycystic kidney disease

## Abstract

Over the last few decades, there has been growing interest in the use of ketogenic diets, mainly as a weight loss strategy. Obesity and diabetes are major risk factors for kidney disease, and obese patients with chronic kidney disease (CKD) are potential candidates for weight reducing diets, among them ketogenic diets. Of further relevance to nephrology, a limited number of recent studies suggests a favorable effect of ketogenic diets in patients affected by autosomal dominant polycystic kidney disease (ADPKD). However, concerns remain about safety in patients with CKD, particularly in the long term and in those with poor residual kidney function. The confusion surrounding the definition of ketogenic diets adds to these concerns. The aim of this review is to summarize information on the main types of ketogenic diets used in daily practice and to discuss the options for their implementation in CKD patients, limiting ultra-processed or industrial preparations.

Although further studies are needed to identify the profile of CKD patients who may benefit from the implementation of ketogenic diets to treat obesity or slow the progression of ADPKD, and to assess short- and long-term safety and adherence, the available data appear promising. This critical review is intended to stimulate further research.

**Graphical abstract:**

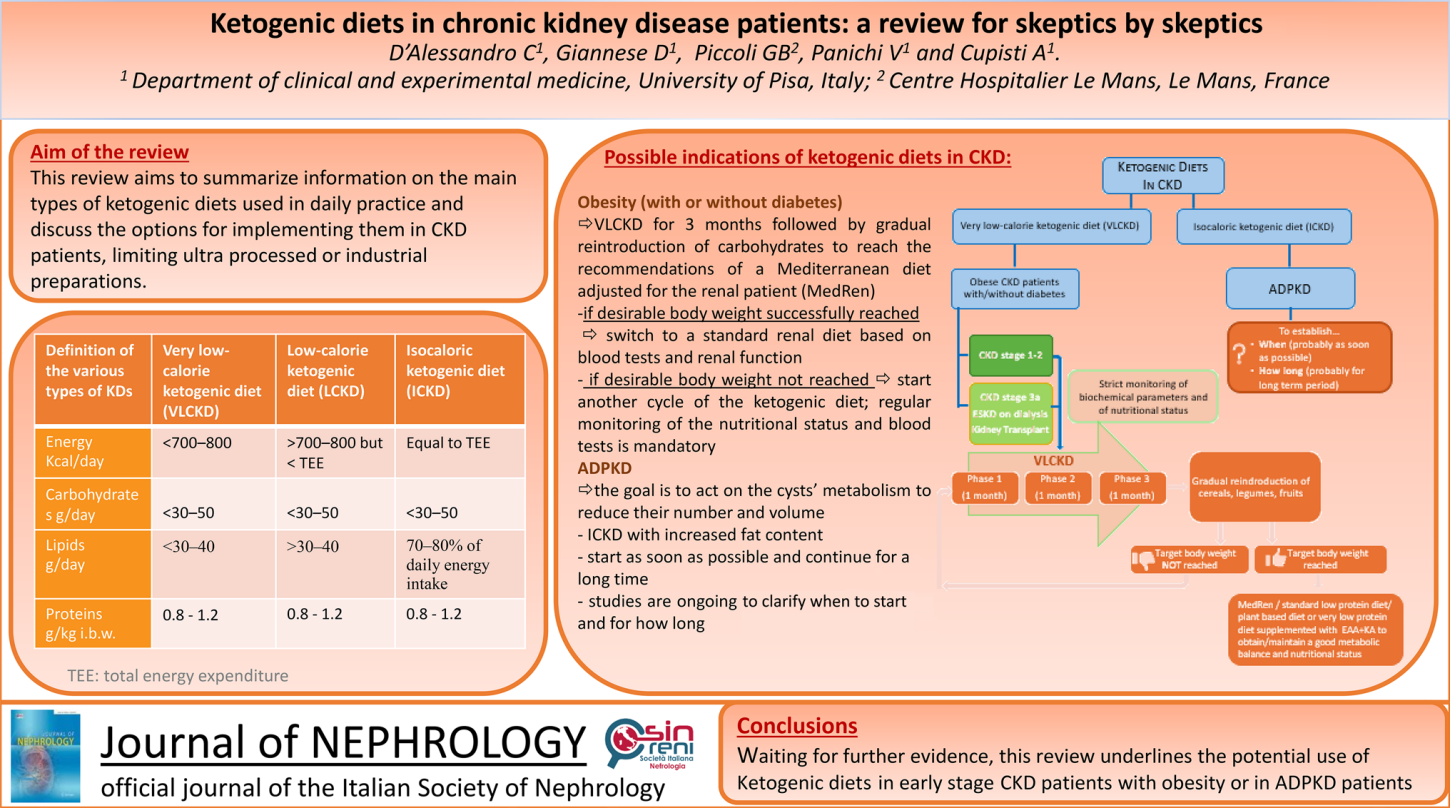

**Supplementary Information:**

The online version contains supplementary material available at 10.1007/s40620-025-02285-7.

## Introduction and historical background

### Ketogenic diets in epilepsy

Ketogenic diet is a term used to define dietary protocols characterized by a drastic reduction in carbohydrate intake and a high fat content. Such a diet induces the production of ketone bodies (acetoacetate, D-3-hydroxybutyrate and acetone) by the liver, which are used as a source of energy [1].

While the use of fasting to treat epilepsy was mentioned in the Corpus Hippocrateum, the first modern observations that “starvation” could be used to treat epilepsy date back to 1911 in Paris, almost synchronous with the observations of Macfadded and his early follower Conklin in the USA, both interestingly practicing what we would now call “alternative or complementary” medicine [2]. While the Parisian doctors limited their experience to epilepsy, their American colleagues claimed that fasting for a minimum of 3 days and for up to 3 weeks could alleviate almost any disease. It was also thanks to another pioneer in the treatment of epilepsy, Dr Geyelin, an endocrinologist at the Presbyterian Hospital in New York, who first published and reported his experience with fasting as a treatment for epilepsy, which also improved cognitive performance, at the American Medical Association Convention in 1921 [3]. At the same time, in 1921 and 1924, Wilder and Peterman at the Mayo Clinic published the results of their studies on the ketogenic diet, which owes its name to Wilder and the first definition of its characteristics to Peterman (for children: 1 g of protein per kg of body weight, 10–15 g of carbohydrates per day and the rest of the calories in fat [4–6].

In the same decade, Cobb and Lennox at Harvard Medical School began a series of studies that resulted in a paper published in 1928 in the New England Journal of Medicine titled “The ketogenic diet in the treatment of epilepsy” [7]. They were the first to report that seizures typically improved after 2–3 days, and Lennox documented that seizure control followed a change in body metabolism induced by fasting or carbohydrate deprivation [7].

While the interest in the ketogenic diet for epilepsy was offset by the introduction of the first effective drugs (with the discovery of the effect of diphenylhydantoin in 1938), interest shifted to weight loss [8].

Notably, interest in ketogenic diet in epilepsy was revived in the early 1990s by a television program and subsequent film (“First Do No Harm”, starring Merryl Streep) based on the true story of Charlie, a 2-year-old boy with intractable generalized seizures, who was seen by Dr Freeman and Ms Millicent Kelly, a nutritionist with specific experience with the ketogenic diet, and rapidly became seizure-free on the diet [9]. Since then, there have been several trials looking at ketogenic diet, initially sponsored by The Charlie Foundation, set up by Charlie’s father [9]. In line with the resurgence of ketogenic diet in epilepsy, several studies are currently underway to evaluate its applications in various diseases, including neurodegenerative diseases such as amyotrophic lateral sclerosis, Parkinson’s disease, Alzheimer’s disease, and in various types of cancer as well as in post-traumatic brain injury [10, 11].

Contrary to what would be expected due to the scarce intake of whole carbohydrates, a positive effect of the ketogenic diets on gut microbiota exists because of the increase of Bacteroidetes to Firmicutes ratio with positive implications in terms of overall inflammation reduction [12, 13]. These results are in contrast with those observed from animal models that associated glucose and lipid metabolism abnormalities to gut microbiota alterations in rats fed ketogenic diets. The changes in microbiota composition were attributed to lower levels of short chain fatty acids and altered composition of cecal bile acids. More studies are needed to investigate not only how this dietary intervention affects host metabolism but also how it indirectly impacts host health through gut microbiota [14].

### Ketogenic diets for weight loss

In the 1970s, Blackburn at Harvard University focused on weight loss following acute events, clearly demonstrating the importance of nutritional status in responding to clinical challenges. Since adding calories or protein was not enough, and lean body mass could only be maintained with different combinations of nutrients, he applied these observations to obesity, where the challenge was similar: maintaining lean body mass during weight loss with very low-calorie treatments, which he defined as protein-sparing modified fasting [15–18].

In 1993, the US Department of Health and Human Services recognized a very low-calorie ketogenic diet as an option for the treatment of obesity, promoting ketogenic diet in campaigns to prevent metabolic diseases. In 2014, the Italian Association of Dietetics and Clinical Nutrition included ketogenic diet in the treatment of obesity and a number of other metabolic diseases [19].

**Fig. 1:**
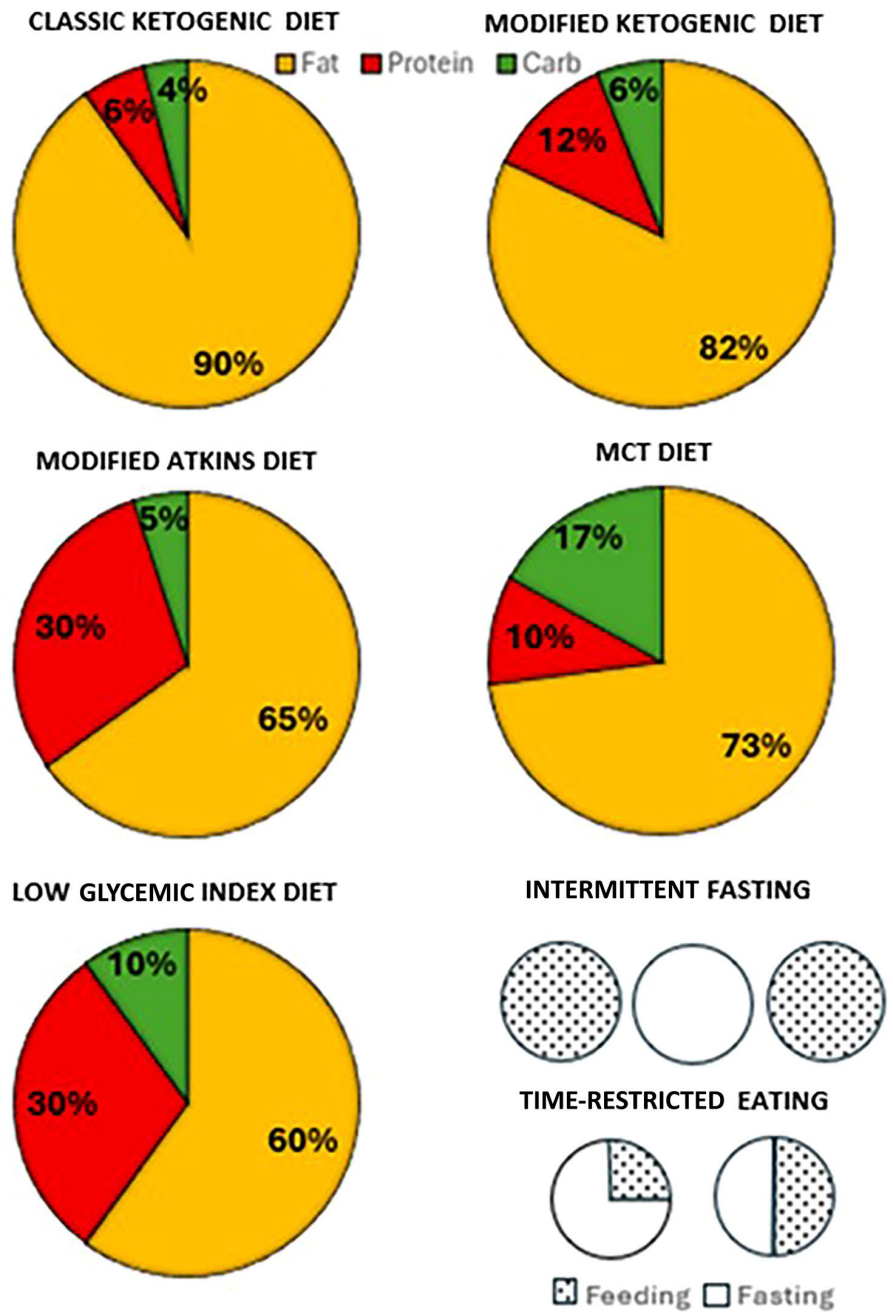
Ketogenic diets. Classic ketogenic diet (KD) provides a ketogenic ratio = 4:1; this means that the amount of fat in the diet must be 4 times higher than that of carbohydrates + proteins. Fats are mainly represented by long-chain triglycerides (LCTs) and represent 80- 90% of daily calories. This diet is used in the neurological field for the treatment of epilepsy; proteins are calculated to meet growth requirements. Modified ketogenic diet is less restricted than the classic keto diet and it can be helpful when starting the diet, it is a more sustainable option for a long-term dietary intervention. Ketogenic ratio ranges from 3:1 to 1:1. Ketogenic diet with medium chain triglycerides (MCT) provides 30-60% of the energy with medium chain triglycerides, which allows to produce more ketones per unit of energy than long-chain triglycerides; this results in a decrease in total fat and a greater intake of carbohydrates and proteins. Ketogenic ratio is around 2:1. Modified Atkins diet is less restrictive than the two previous types, as it allows a carbohydrate intake of up to 20 g/day; the ketogenic ratio is around 1:1 and is usually intended for the adult population. Low glycemic index diet allows a carbohydrate intake of 40-60 g/day, limited to foods with a low glycemic index (GI < 50). It is the least restrictive of these four diets, but also the one that produces lower levels of circulating ketones. The ketogenic ratio is 2:3. Intermittent fasting consists of alternative days of fasting and ad libitum feeding. The restricted time feeding consists of a daily restriction of feeding window

A crucial aspect is that the dietary protocols used to induce weight loss are different from those used in the treatment of epilepsy (see next paragraph for more details). Despite the shared problem of ketone production, the ketogenic diets used in the treatment of obesity usually have a reduced energy intake (from 800 to 1200 kcal per day), whereas the diets used in the treatment of epilepsy are isocaloric with a high lipid content (Fig. 1; Table 1).

**Table 1:** Definition of the various types of ketogenic diets. Modified from Trimboli and coworkers [20]

Diet	Energy Kcal/day	Carbohydrates g/day	Lipids g/day	Proteins g/kg i.b.w
Very low-calorie ketogenic diet	< 700–800	< 30–50	< 30–40	0.8–1.2
Low-calorie ketogenic diet	> 700–800 but lower than total energy expenditure	< 30–50	> 30–40	0.8–1.2
Isocaloric ketogenic diet (ICKD)	According to Total energy expenditure	< 30–50	70–80% of daily energy intake	0.8–1.2

The energy intake is up to 700–800 kcal/day in Very low-calorie ketogenic diet (VLCKD), it is a fixed value and not established based on body weight. In Low-calorie ketogenic diet (LCKD) and Isocaloric ketogenic diet (ICKD), instead, the energy intake should be calculated on an individual basis depending on the energy needs. Protein intake should not be increased to over 0.8–1.2 g/kg because this could determine the activation of gluconeogenesis thus inhibiting ketosis. For this reason, the increase in energy content in the LCKD and ICKD can only be obtained with an increase in lipid intake

*VLCKD* Very low-calorie ketogenic diet; i.b.w.: ideal body weight, *LCKD* Low-calorie ketogenic diet, *ICKD* Isocaloric ketogenic diet

In nephrology, two main applications of ketogenic diet are currently identified: weight loss, with different objectives according to the stage of chronic kidney disease (CKD), namely in the early stages of CKD to stabilize kidney function and control proteinuria, in the late stages of CKD mainly to allow access to kidney transplantation, and, more recently, the treatment of autosomal dominant polycystic kidney disease (ADPKD), the latter being a matter of some concern in our field [20–22]. Therefore, this narrative critical review was planned to analyze the available evidence, the knowledge gaps and the barriers to the implementation of ketogenic diet in nephrology, as a tool to support those who are skeptical, but who have to deal with an increasing number of patients opting for this treatment.

## The Ketogenic diet: what it is, and what it is not

Table 1 summarizes the definitions of the different types of low-calorie ketogenic diets recently proposed by Trimboli [23]. The energy intake is up to 700–800 kcal/day in a very low-calorie- ketogenic diet, it is a fixed value and is not based on body weight. In both low-calorie- ketogenic diet and isocaloric- ketogenic diet, however, energy intake should be calculated on an individual basis according to energy requirements.

**Fig. 2:**
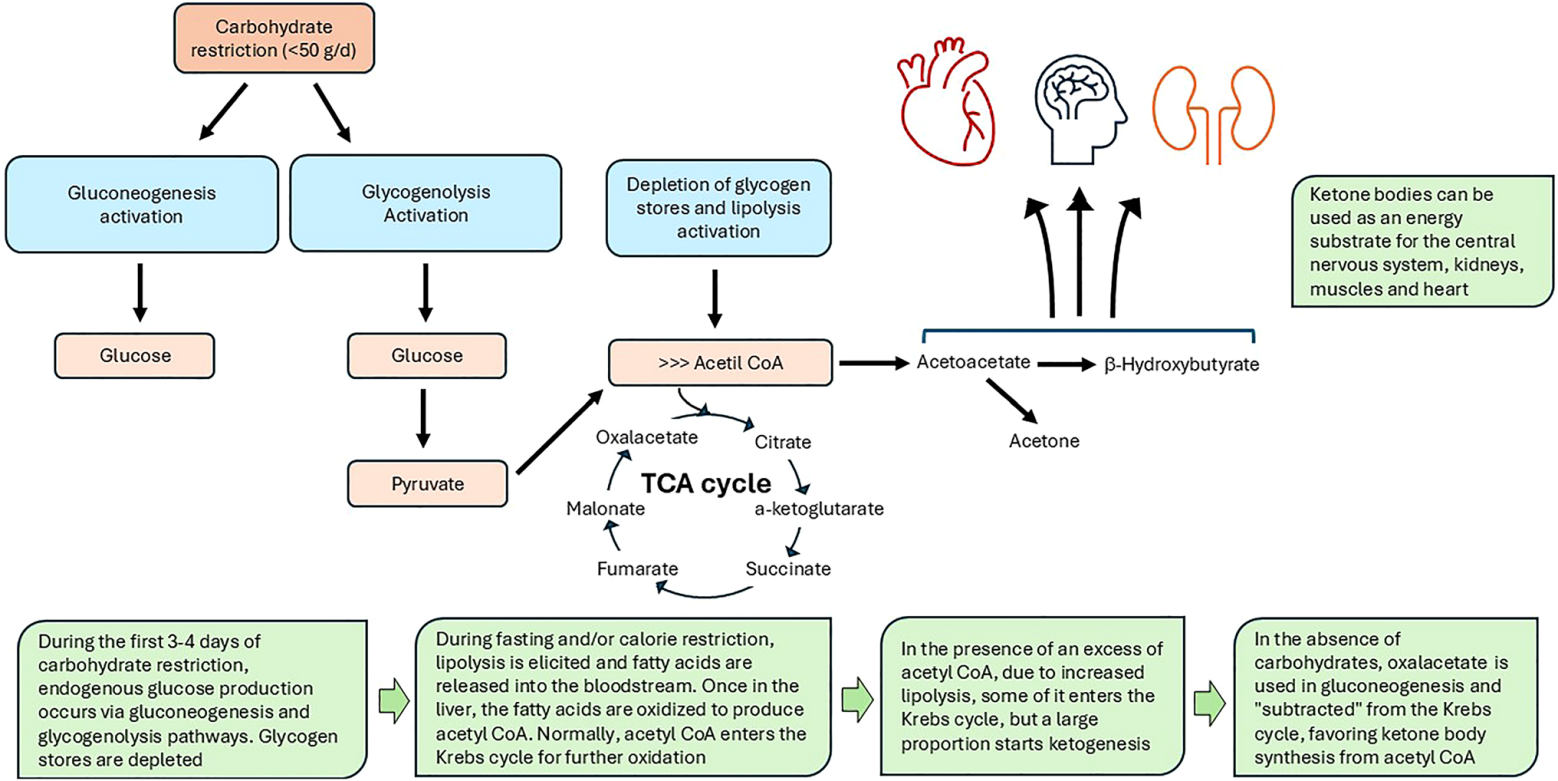
Mechanism of ketogenesis. Details are given in the green boxes and in the text

In general, to be defined as ‘ketogenic’, the diet must not exceed 30–50 g of carbohydrate per day. Figure 2 summarizes the mechanism of ketogenesis. During the first 3–4 days of carbohydrate restriction, endogenous glucose production occurs via gluconeogenesis and glycogenolysis pathways. In this state, glycogen stores are depleted and in the absence of glucose, the cell has to find alternative fuels for energy production, namely ketone bodies. Ketones are produced from acetyl-CoA. During fasting and/or calorie restriction, lipolysis is triggered and a large amount of fatty acids are released into the bloodstream. Once in the liver, the fatty acids are oxidized to produce acetyl CoA. Normally, this compound enters the Krebs cycle for further oxidation. In the presence of an excess of acetyl CoA, resulting from the large amount of fatty acids released by lipolysis and oxidized in the liver, some of it enters the Krebs cycle, but a large proportion triggers ketogenesis. Furthermore, in the absence of carbohydrates, oxalacetate (an intermediate in both the Krebs cycle and gluconeogenesis) is used in gluconeogenesis and “subtracted” from the Krebs cycle, further favoring the entry of acetyl CoA into the synthesis of ketone bodies [24]. The latter can be used as an energy substrate for the central nervous system, kidneys, muscles and heart. Ketogenic diets induce a condition called “nutritional ketosis” [25]. Nutritional ketosis is characterized by a blood ketone level between 0.5 and 3 mg/dl. However, it has been reported that to obtain a therapeutic effect, the ketone concentration should ideally be higher, between 5 and 8 mg/dl: this is called “therapeutic ketosis”. The therapeutic effect is mediated by several mechanisms, including reduction in blood glucose, glycated hemoglobin and serum insulin levels, improvement in insulin sensitivity, reduction in hunger and reduction in inflammation [26].

Once the state of ketosis is achieved, the human body does not optimally use ketone bodies as fuel but requires specific adaptations to switch from glucose to ketone bodies as an energy source [27]. This process has been termed ‘keto adaptation’ and is due to up-regulated transcription of genes encoding metabolic enzymes leading to increased mitochondrial density in oxidative tissues such as muscle and brain [25, 28].

During this period of adaptation, the body maintains normal pH and blood glucose levels.

This is the main difference from diabetic ketoacidosis, a pathological condition in which ketone body levels can be five to ten times higher than in nutritional ketosis and which, if not treated promptly, can lead to coma and even death.

## Ketogenic diet to treat obesity

Table 1 shows that energy intake for low-calorie ketogenic diet and isocaloric-ketogenic diet is defined based on the patient’s total energy expenditure, whereas energy intake for very low-calorie ketogenic diet is fixed (approximately 800 kcal/day),

Protein intake is targeted at approximately 0.8–1.2 g/kg of ideal body weight, so that in overweight or obese individuals on very low-calorie- ketogenic diet, the protein intake per kg of actual weight is reduced [29].

Very low-calorie- ketogenic diet is usually implemented using industrial products in the form of milk shakes or bars, or ready-to-cook or ready-to-eat meals (supplementary Table 1). These ‘artificial meals’ are based on high biological value proteins derived from soy, green peas, whey and eggs, and provide approximately 100–150 kcal per meal. The pre-packaged products used in a dietary supplement are often presented in forms that only require reheating and/or are ready to eat and prepare, such as bars or vanilla, caramel, chocolate or flavored powders. They generally have a high protein content, even though the main characteristic of a ketogenic diet is a low carbohydrate and high fat intake. These industrial products may contain unhealthy saturated fats and are usually fortified with trace elements and vitamins (supplementary Table 1) and preservatives. The cost of a diet based mainly on these products can be considerable.

The weight loss program is usually divided into different phases (Table 2). The first three represent the ketogenic period, which may last 8–12 weeks and can be prolonged until 80–85% of the target weight loss has been achieved. The next phases are characterized by the gradual reintroduction of different food sources of carbohydrates, selected on the basis of glycemic index and energy content, until a healthy maintenance diet is reached, as recommended by the WHO, with a balanced ratio between macronutrients, i.e. 55% carbohydrates, preferably complex, 25–30% fats, mainly of vegetable origin, and the remaining part in proteins, preferably of vegetable origin [30, 31].

**Table 2:** Planning of a very low calorie ketogenic diet (VLCKD) (modified from Caprio et al. 2019) [40]

Ketogenic phases (8–12 weeks)			Maintenance phase		
Phase 1	Phase 2	Phase 3	Phase 4	Phase 5	Phase 6
4–6 artificial meals + low carbohydrate vegetables	3–5 artificial meals + low carbohydrate vegetables + 1 meal with regular food source of protein (meat/fish/eggs/soy)	3–5 artificial meals + low carbohydrate vegetables	Gradual reintroduction of carbohydrates with low glycemic index (fruit and dairy products)	Reintroduction of foods with moderate glycemic index (legumes) in addition to changes made in phase 4	Reintroduction of foods with high glycemic index (bread, pasta) in addition to changes made in phase 5
600–800 kcal			800–1500 kcal		

Regular physical activity is recommended from phase 4 (Table 2). Close medical supervision and monitoring of biochemical parameters is highly recommended [32].

## Potential applications of the ketogenic diets in nephrology: cardiovascular risk

The 2014–2016 guidelines for the management of obesity of different societies, namely the American Heart Association, the American College of Cardiology, the Task Force on Practice Guidelines and The Obesity Society, the National Institute of Health and the UK Nice suggest using a very low-calorie ketogenic diet in patients who failed losing weight with conventional low calories diets [33, 34].

However, in 2023, the American Heart Association subsequently published a new statement to outline the main features of heart-healthy dietary patterns; in this document, the ketogenic diet was rated as the least favorable in preventing cardiovascular diseases [35]. The reason is that grains, legumes, fruit and vegetables, and dairy products are sharply reduced or banned while the content of animal-derived food and saturated fats is comparatively high, a profile known to increase cardiovascular risk. However, studies on the effects of a ketogenic diet on cardiovascular diseases in humans yielded mixed results. For example, the ketogenic diet increases low-density lipoprotein cholesterol but decreases triglycerides [36]. Indeed, the American Heart Association statement highlighted that some studies have shown improvements of cardiovascular risk factors, including body weight, blood glucose, high density lipoprotein (HDL) cholesterol and triglycerides at 6 months, but statistical significance versus control diets disappeared at 12 months [35, 37].

## Ketogenic diets in CKD

The benefits of dietary management, particularly the use of well-designed and controlled low-protein and Mediterranean diets in CKD, are widely recognized [38, 39]. A stepwise approach or simplified dietary regimens are usually prescribed as part of a personalized approach that takes into account not only residual kidney function and progression, but also socioeconomic, psychological and functional aspects, and patient preferences [40–42]. Based on the main characteristics of CKD, there are some concerns regarding its implementation in patients with CKD, especially in those with severe decline in kidney function. Namely, the increased net acid load and high dietary protein intake may exacerbate metabolic acidosis, induce hyperkalemia, protein catabolism and accelerate the progression of kidney damage. Ketones are acidic, and an increase in blood levels leads to a reduction in the alkaline reserve, resulting in metabolic acidosis [1]. However, in nutritional or therapeutic ketosis, the body appears to be able to maintain a normal pH, suggesting that the risk of metabolic acidosis is low, at least in the early stages of CKD [25]. As mentioned above, protein intake is not necessarily increased and its source is not restricted; however, there are concerns if the diet is mainly based on commercial (often ultra-processed) products.

### Contrasting obesity in CKD

However, reported experience is limited and heterogeneous. Suyoto et al., in a meta-analysis of 12 randomized controlled trials involving 942 obese patients with type 2 diabetes, 500 of whom on very low-calorie ketogenic diet and 442 on a control diet, found no adverse effect on kidney function in patients on very low-calorie ketogenic diet compared with the control group [43]. In a retrospective cohort study, Mitchell included adult patients with CKD stages 1 to 3, with or without diabetes, and showed that estimated glomerular filtration rate (eGFR) was stable or improved in patients with CKD stages 2 and 3 on ketogenic diet , regardless of diabetes [44]. In non-diabetic patients with stage 3 CKD, greater weight loss was associated with an improvement in eGFR, whereas in patients with stage 1 CKD, eGFR decreased by approximately 4-6 ml/min due to a reduction in hyperfiltration. The improvement in eGFR in stage 2-3 was attributed to rapid weight loss, which led to better control of blood pressure and glucose [44].

Friedman et al. studied the effects of ketogenic diet in six obese subjects with advanced diabetic nephropathy (eGFR <40 ml/min per 1.73 m^2^, urinary albumin excretion >30 mg/d) [45]. Patients received a very low-calorie- ketogenic diet (800 kcal/d, carbohydrate <50 g/d and about 75 g protein) with four commercial supplements (two 15 g protein bars and two shakes/smoothies/soups, one every 2–3 hours), one lean meal (two servings of vegetables and one rich in protein), and a multivitamin supplement, and were encouraged to drink at least 2 liters of water or non-caloric beverages. Over the 12-week study period, the authors observed a median weight loss of 12%, a 36% reduction in albuminuria (2124 vs 1366 mg/24 h, *p*=0.08), reductions in serum creatinine (3.54 vs 3.13 mg/dl, *p*<0.05) and cystatin C (2.79 vs 2.46 mg/l, *p*<0.05), without changes in serum urea levels. There were also reductions in fasting glucose (166 versus 131 mg/dl, *p*<0.05), fasting insulin (26.9 versus 10.4 μU/ml, *p*<0.05) and insulin resistance (9.6 versus 4.2, *p*=0.03). The biochemical changes were accompanied by a significant improvement in physical function and general health, and a reduction in antidiabetic medication. A transient increase in serum creatinine and urea levels was observed at the start of ketogenic diet , which was corrected by reducing antihypertensive medication [45].

The very low-calorie ketogenic diet has also been used in patients with kidney failure on dialysis. Lassemillante et al. reported on 5 hemodialysis (HD) patients whose weight loss allowed them to wait for kidney transplantation [46]. The diet (950 kcal and 100 g protein per day) included three meal replacements, one meal based on a “normal” food source of protein, and two fruits per day, selecting those with the lowest potassium content. The results showed a median weight loss of 7% (range 5.2-11.4%) with no significant side effects. The authors concluded that the modified low-calorie diet was safe and effective and that meal replacements could be an alternative weight loss strategy in HD patients [46]. Woods et al. reported on 22 hemodialysis patients (9 on home hemodialysis, 13 on in-center hemodialysis) with a body mass index (BMI) > 30 kg/m2 who followed a modified very low-calorie- ketogenic diet (600-800 kcal/day) for 12 weeks, with weekly monitoring for the first 4 weeks and then every 2 weeks until the end of the study [47]. Pre-dialysis body weight decreased by an average of 0.91 kg per week (95% C.I. 0.74 - 1.08, p<0.001). Patients on home hemodialysis achieved the best results in weight loss (1.13 kg per week, 95% C.I. 0.89 - 1.37). At the end of follow-up, waist circumference was reduced by an average of 10.5 cm (95% C.I. 7.4 - 13.6, p<0.001). Nine patients were eligible for kidney transplantation. One episode of hyperkalemia (K> 6.0 mEq/l) was recorded, and potassium dialysate concentration was reduced in 6 patients [47].

In 2000, Lambert et al. developed a guideline for the use of very low-calorie- ketogenic diet in obese CKD patients, both pre-dialysis and on dialysis [48]. The protocols used, which are summarized in supplementary Table 2 , are based on industrial meal replacements (shakes, bars) as used in many settings. In dialysis patients, energy and protein intakes are slightly higher than in CKD stages 1-4. Notably, patients in stage 5 who are not on dialysis are not included because of the risk of clinical instability [48].

Based on their experience, the authors suggest the following indications:

- Patients with CKD stage 1–2 and BMI > 30 kg/m^2^, especially with type 2 diabetes;
- Patients with CKD stage 3–4 not on dialysis and BMI > 30 kg/m^2^ or increased waist circumference who are potential candidates for kidney transplantation;
- Patients with CKD stage 5D and BMI > 30 kg/m^2^ who are potential candidates for kidney transplantation; or patients who may benefit from weight loss for other reasons, e.g. to improve mobility or reduce joint pain.

Of note, the authors suggest a monitoring schedule and identify some potential dietary drug interactions [48].

In a recent controversy, Weimbs and Joshi discussed the pros and cons of ketogenic diet in CKD [49, 50]. Weimbs uses the term “ketogenic metabolic therapy” rather than “ketogenic diet” as they propose this dietary intervention as a medical nutrition therapy for some CKD-related health conditions such as obesity, metabolic syndrome and ADPKD; the authors emphasize that there is no need for a large increase in animal protein in such a diet and strongly support a plant-based version of ketogenic diet [49]. Conversely, Joshi discusses the limitations of the evidence, the problem of long-term compliance and the risk of kidney stones; while stressing the problem of increasing unhealthy fats of animal origin, the authors leave some room for a plant-based ketogenic diet in CKD patients [50]. In Italy, Zoccali et al. proposed a multicenter randomized controlled trial (RCT) protocol to evaluate the effect, benefits and harms of a dietary intervention in patients with mild-to-moderate non-diabetic CKD and mild-to-severe obesity [51]. The primary outcome is weight loss at 6 months, with secondary outcomes including dietary adherence, changes in body composition, blood pressure, lipid profile, biomarkers of mineral bone disease and kidney function. The proposed diets are ketogenic diet (usual energy intake: ∼fat 70%, protein 25%, carbohydrate 5%); control diet (0.8 g/kg/day protein, ∼fat 25%, carbohydrate 60%) [51]. The interest aroused by this study shows that, at least in Italy, these issues are considered very important in the context of medical nutrition therapies. Demonstrating this growing concern, very recently, The Italian Society of Endocrinology, the working group of the Club Nutrition – Hormones and Metabolism, the Italian Society of Nutraceuticals, Club Ketodiets and Nutraceuticals “KetoNut SINut” and the Italian Society of Nephrology produced a consensus statement to support Endocrinologists, Dietitians, Nutritionists and Nephrologists with practical guidance for the management of obesity in CKD patients [52].

### Autosomal dominant polycystic kidney disease and ketogenic diet

In ADPKD, one of the most common genetic diseases affecting the kidneys, altered cell proliferation and abnormal activation of mTOR signaling pathways, defective glucose metabolism, dysregulation of lipid and amino acid metabolism, abnormal apoptosis mechanism and mitochondrial dysfunction have been reported; some of these alterations seem potentially amenable to nutritional interventions, besides increasing water intake [53–57].

The alterations in glucose metabolism have encouraged consideration of dietary manipulation and the use of metformin, which appears to have some effect in animal models, but with inconsistent results in humans [58]. Alteration of the mTOR pathway is characterized by increased glycolysis and mitochondrial dysfunction with decreased oxidative phosphorylation in animal models, leading to the hypothesis of a Warburg effect like that observed in cancer cells [59]. This was supported by the observation that treatment of 1 (PKD1)-deficient mice with an inhibitor of glycolysis, namely 2-deoxiglucose, resulted in a reduction in cyst growth [60].

The sensitivity of mTOR pathways to nutrient availability provided the basis for animal models. Kipp et al. observed that a moderate reduction in food intake (about 20%) slowed disease progression in a mouse model through mTOR modulation [61]. Warner et al. found that a 40% reduction in food intake from 6 weeks to 7.5 months was associated with a 50% reduction in kidney size, cystic index, inflammatory infiltrates and fibrosis in PKD1-deficient mice [62]. In a rat model, Torres showed that ketogenic diet and time-restricted feeding without calorie reduction strongly inhibited mTOR signaling, proliferation and fibrosis in the affected kidneys and even led to a regression of renal cystic load [63].

The explanation is that cyst cells are unable to use ketone bodies as fuel. In addition, Torres et al. found that PKD rats treated with beta-hydroxybutyrate for five weeks showed reduced disease progression and kidney fibrosis. The same effects were also found in PKD rats fed a high-carbohydrate diet. The authors suggested that inducing a state of ketosis, either by dietary intervention or by mimicking its effects with beta-hydroxybutyrate, could be an effective treatment for ADPKD in humans [63–65].

Strubl et al. retrospectively evaluated the feasibility, safety and effects of ketogenic diets in 131 ADPKD patients, 74 of whom had followed a ketogenic diet for at least 6 months [66]. According to patient reports, 86% of participants felt that their general health had improved, 67% described an improvement in symptoms, 64% of hypertensive patients reported a reduction in blood pressure and 90% reported significant weight loss. However, 66% of patients reported side effects commonly associated with ketogenic diet, namely ‘keto flu’, fatigue and hunger; overall, 92% found ketogenic diet feasible [66].

Among the many recent studies, a few are worth mentioning: Testa et al. conducted a pilot study to evaluate the safety and tolerability of a modified Atkins diet in ADPKD patients using the Modification of Diet in Renal Disease Study Dietary Satisfaction Questionnaire, a 29-item questionnaire with answers ranging from 1, the worst experience, to 5, the best experience. They found good results in terms of satisfaction, adherence and reported well-being (median score 4, IRQ 4–5; 3, IRQ 2–5; 5, IRQ 4–5), as well as a reduction in blood glucose levels. An increase in cholesterol levels was observed [21]. The same group is currently conducting a phase II trial of the modified Atkins diet in patients with rapidly progressive ADPKD [22]. Conversely, to test whether a short-term dietary intervention is feasible and has some carry-over effect, RESET-PKD, a short-term pilot study involving 20 patients with rapidly progressive disease, proposed starting with a carbohydrate-rich diet, followed by randomization to a 3-day water fast or a 14-day ketogenic diet. At the third visit, patients resumed their usual diet for 3–6 weeks. While 9 out of 10 patients achieved a ketogenic state and rated the diet as feasible, total kidney volume did not change during this short-term trial, while total liver volume was significantly reduced [67].

In an attempt to compare different options, Cukoski et al. presented the results of a 3-month clinical trial involving 66 ADPKD patients who were randomized into three groups: one group following a low-carbohydrate, high-fat ketogenic diet, one group following a 3-day water fast once a month, and a control group that received routine advice for ADPKD patients [20]. The authors observed that both ketogenic diet and water fasting induced ketogenesis, with a significant reduction in fat mass and liver volume with ketogenic diet and a reduction in kidney volume that did not reach statistical significance. The authors suggested that this reduction could be due, at least in part, to glycogen depletion as observed during caloric restriction. eGFR (calculated on both creatinine and cystatin C) decreased in the control and water fasting groups but increased in the ketogenic diet group. The change in the ketogenic diet group was statistically significant. Overall, 95% of patients in the ketogenic diet group and 85% in the water fasting group considered the diet feasible. Flu-like symptoms and fatigue were reported in the first 2–7 days of ketogenic diet [20].

One patient showed a symptomatic kidney stone on ketogenic diet. The authors claimed that it was not possible to state whether this was due to the diet or not, but it can be considered a plausible connection considering the high prevalence of kidney stones in ADPKD and the increase in uric acid and kidney stone formation observed in epilepsy patients on ketogenic diet [20].

In any case, a point to be stressed is that ketogenic diets (both low or very low-calorie ketogenic diet used to treat obesity or classic ketogenic diet, and its modification proposed for ADPKD patients) are not high in protein and this mitigates the risk of stone formation. Nevertheless, potential preventive measures should be considered, such as alkali supplementation, based on urine profile.

## Ketogenic diet, CKD, substitute meals and options for a “natural” implementation

In the few studies available on CKD patients, very low-calorie- ketogenic diet is mainly implemented using industrial products. Although their use simplifies the diet, not all patients like them, the cost is high and the use of processed foods is contrary to the latest approaches to “renal diets”.

Given that, at least in our setting, patients are increasingly requesting or choosing to start a ketogenic diet, the development of plans using natural foods, minimizing the use of artificial products and controlling protein content should be further explored, also in line with the recent controversy on these issues [49, 50].

Table 3 provides some examples of a 3-stage ketogenic diet plan using natural foods. Table 4 suggests food choices to vary the diet without affecting ketogenesis, considering the limitations of phosphorus (and possibly potassium) in CKD patients.

**Table 3:** Very low calorie ketogenic diet (VLCKD) implementation with natural foods

Phase 1 (1 month)		Phase 2 (1 month)		Phase 3 (1 month)	
Breakfast	Amount, g	Breakfast	Amount, g	Breakfast	
Bread	30	Bread	30	Bread	30
Avocado	30	Avocado	30	Avocado	60
Snack		Snack		Snack	
Yogurt	70	Yogurt	100	Yogurt	120
Nuts	15	Nuts	15	Nuts	15
Lunch		Lunch		Lunch	
Konjac pasta*	50	Konjac pasta*	50	Konjac pasta*	50
Meat (veal)	60	Meat (veal)	100	Meat (veal)	90
Vegetable (fennel)	100	Vegetable (fennel)	100	Vegetable (fennel)	150
Olives	10	Olives	10	Olives	20
Olive oil	10	Olive oil	15	Olive oil	20
Dinner		Dinner		Dinner	
Konjac pasta*	50	Konjac pasta*	50	Konjac pasta*	50
Fish (cod)	70	Egg	55–60 (1 egg)	Fish (cod)	100
Vegetables (chard)	100	Vegetables (chard)	100	Vegetables (chard)	150
Parmesan cheese	20	Parmesan cheese	20	Parmesan cheese	20
Butter	10	Butter	10	Butter	15
Olive oil	10	Olive oil	15	Olive oil	20
Nutritional facts		Nutritional facts		Nutritional facts	
Energy, Kcal	877	Energy, Kcal	1099	Energy, Kcal	1320
Protein, g	43.4	Protein, g	49.1	Protein, g	60.1
Fats, g	64.4	Fats, g	85.5	Fats, g	104
Carbohydrates, g	26.8	Carbohydrates, g	29.3	Carbohydrates, g	30.4
Sugars, g	6.9	Sugars, g	8.9	Sugars, g	10.0
Fiber, g	20	Fiber, g	21.6	Fiber, g	24
Phosphorus, mg	633	Phosphorus, mg	754	Phosphorus, mg	857
Sodium, mg	772	Sodium, mg	984	Sodium, mg	1002
Potassium, mg	1400	Potassium, mg	1700	Potassium, mg	2130
Protein, %	20.3	Protein, %	18.2	Protein, %	18.6
Fats, %	67.9	Fats, %	71.5	Fats, %	72.6
Carbohydrates, %	11.8	Carbohydrates, %	10.2	Carbohydrates, %	8.8

Examples of dietary plans with natural foods. Phase 3 is followed by a gradual reintroduction of cereals and fruit according to the principles of the Mediterranean diet adjusted for the renal patient accompanied by adequate physical activity [33]. If the desired weight loss is not reached, it is possible to start again from phase 1 after checking the patient’s blood tests and carrying out nutritional status assessment ^*^Konjac pasta is made of glucomannan, a soluble fiber. Its use in a Ketogenic diet is addressed in order to increase satiety but it is optional; it does not interfere with energy content or with the ketogenic process

**Table 4:** Some examples of food choices to implement a Ketogenic Diet in CKD using natural food

Food	Ketogenic diet features	Concerns and suggestions for CKD patients
Nuts and derivatives (almond, macadamia nuts, peanuts, hazelnuts, almond flower)	Rich in unsaturated fats and fiber	High potassium and phosphorus content: respect suggested amount
Fruit, seeds and derivatives		
Group 1: Avocado, olives, coconut, Coconut flower, pumpkin seeds, flax seeds, sunflower seeds, cocoa, dark chocolate (85–90%)	Rich in fats	High potassium and phosphorus content: respect suggested amount
Group 2: courgettes, fennel, aubergines, mushrooms, lettuce, cucumbers	< content of carbohydrate Rich in fiber	Vegetables are rich in potassium: boiling helps in reducing its content
Group 3: spinach, cauliflower, broccoli, asparagus, tomatoes, pumpkin, peppers, beets, artichokes	< content of carbohydrate Rich in fiber	
Meat, fish and derivatives		
Any type of meat and fish. Fatty fish like fresh salmon and mackerel are to be preferred	High content of (saturated) fats and cholesterol	The dietary plans proposed in Table 3 have a reduced content of energy, therefore the servings are inevitably small For the same reason phosphorus content is also low Preserved meat consumption should be avoided due to the high content of salt and phosphorus from additives Respect suggested amount to control cholesterol intake
Milk and derivatives		
Full fat Greek yogurt, mascarpone cheese, gorgonzola cheese, spreadable cheese, butter, ghee, cream	High content of (saturated) fats and cholesterol	Cheese is rich in phosphorus and salt. Spreadable cheese may have preservatives Respect suggested amount to control cholesterol intake
Eggs	Source of protein of high biological value	High phosphate and cholesterol content: Respect suggested quantities or use only egg white
Konjac pasta	Source of fiber, no carbohydrates, no calories	No contraindications in CKD. Adverse effects could be swelling and abdominal distension if consumed in large amounts

*The standard ketogenic diet recommends that the use of preserved meats such as mortadella, salami, sausages, bacon should be avoided in CKD patients due to the high salt content and presence of preservatives

Suggestions are given on to how to prepare recipes with the allowed ingredients

In the early days of a low-calorie CKD diet, patients may feel hungry, but this will subside once ketosis is established. At this stage, the use of Konjac pasta could be an interesting option. This product, available in most supermarkets, is made from glucomannan, a soluble fiber extracted from the root of a plant called konjac, which is widespread in Asia [68]. As a fiber, it is not absorbed and does not provide calories or affect ketogenesis; it absorbs water and increases its volume by about 60 times, producing a long-lasting feeling of satiety. It is a source of fiber that compensates for the low intake of ketogenic diet.

Regular monitoring of blood tests and body composition is, of course, necessary. The third phase is a gradual reintroduction of cereals, fruits and legumes to restore a healthy and balanced diet. Once the target body weight has been reached, further adjustments should be considered, depending on residual kidney function [38–41].

The very low-calorie-ketogenic dietary plans here proposed are low in calories, the percentage of fat is high, but the absolute amount of protein is not as high as we might expect for a very low carb diet to obtain a reduced energy intake (Table 3). This could lead to the risk of deficiency of essential amino acids and of protein malnutrition. Hence, we could speculate on the possibility of supplementation with essential amino acids and ketoacids (EAA + KA), which is used in low protein and very low protein regimens in advanced CKD, whose efficacy and safety have been widely proven [38, 69, 70].

Supplementary Table 3 summarizes possible side effects of very low-calorie- ketogenic diet in CKD patients, and provides some suggestions for their management, drug adjustment and monitoring.

**Fig. 3:**
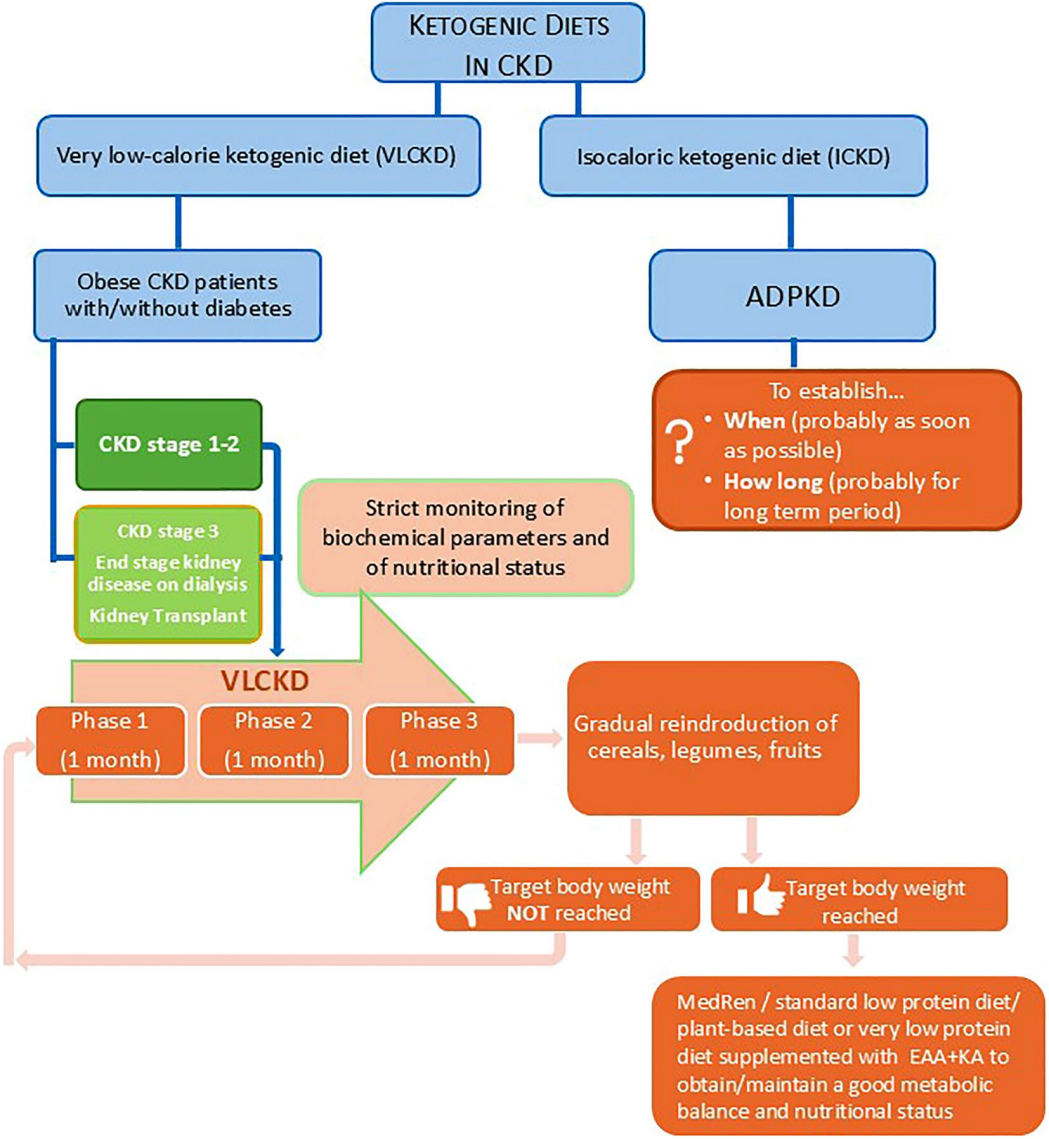
Possible applications of the ketogenic diets in CKD. Obesity and diabetes. In this case, VLCKD diets are used for a total of 3 months, then a gradual reintroduction of sources of carbohydrates (cereals, fruit and legumes) is carried out until the recommendations of a Mediterranean diet adjusted for the renal patient (MedRen) are reached. If the desirable weight has been reached, we evaluate whether to continue with MedRen or, if necessary, switch to a standard renal diet based on blood tests and renal function. If the desirable body weight has not been reached, start another cycle of the ketogenic diet, accompanied by regular monitoring of the nutritional status and blood tests. ADPKD. In this case the goal is to act on the cysts’ metabolism to reduce their number and volume. The isocaloric ketogenic diet with increased fat content, probably should start as soon as possible and continue for a long time. Stud are ongoing to clarify these issues

Figure 3 proposes a flow chart for the potential use of ketogenic diet both in the case of obese CKD patients and for patients with ADPKD.

## Ketogenic diet, CKD, electrolytes and hydration

Ketogenic diets are considered at risk of electrolyte imbalances. The limited intake of vegetables and fruit with a ketogenic diet is considered a trigger for hypokalemia [71]. Moreover, low-fat and low-calorie diets are associated with a reduction in renin and aldosterone levels [72]. Increased sodium excretion is observed in ketogenic diets, leading to increased renal salt and water loss. This volume reduction may start compensatory aldosterone secretion to preserve blood volume and reduce salt excretion. The impact of ketogenic diets on aldosterone secretion has not been thoroughly investigated, nor has the effect of salt supplementation, however, in the implementation of ketogenic diets, salt and mineral supplementation is recommended together with an increase in fluid intake. Belany et al. in a two-arm RCT, examined the impact of two ketogenic diets, one with and one without an exogenous ketone salt supplement [73]. The study provides valuable information on dietary regulation of RAAS activity and the links between ketosis and aldosterone regulation. As expected, nutritional ketosis led to an increase in aldosterone levels but, unexpectedly, the increase was greater in subjects supplemented with sodium chloride and magnesium-β-hydroxybutyrate salts. This was associated with a reduction in renin plasma levels, suggesting that during nutritional ketosis aldosterone production is not associated with volume depletion and increased salt excretion, but it is probably independently regulated. Nutritional ketosis and exogenous salt exposure seem to play an additive role in increasing aldosterone levels [74].

CKD patients are generally recommended to limit salt and preserved foods intake, avoid mineral salt supplements to prevent fluid retention and an increase in blood pressure, and to reduce the risk of hyperkalemia. Therefore, in the case of a patient with CKD undergoing a ketogenic diet, the “a priori” suggestion to integrate mineral salts or increase water intake must be carefully evaluated. Regular monitoring of fluid and ion status is mandatory to evaluate the need for specific supplements or to prescribe the amount of fluid intake.

## Conclusions

The use of ketogenic diet in patients with CKD represents a new option and a challenge for nephrologists and dietitians. The heterogeneity of the trials makes it difficult to compare the still limited results. However, the risks are likely to be lower than expected and ketogenic diet can be used safely in the treatment of obesity when managed by skilled operators and for limited periods of time. However, commercial industrial products may be an inappropriate choice for CKD patients, both because of the composition and the high cost, but the possibility of designing a ketogenic diet with ‘normal’ food exists and should be pursued.

While awaiting further evidence, the authors of this review emphasize the importance of responding to the increasing demands of CKD and ADPKD patients. The type of ketogenic diet for obese CKD patients is different from that for ADPKD, although they share the mechanism of ketosis. Patients should be warned that ketogenic diet is feasible but requires close monitoring for safety.

We eagerly await data from ongoing studies of the effects of ketogenic diet in kidney patients to define who can most benefit from this treatment, when to start and how to continue it over time.

## Supplementary Information

Below is the link to the electronic supplementary material.

Supplementary file1 (DOCX 312 KB)

Supplementary file2 (DOCX 19 KB)

Supplementary file3 (DOCX 21 KB)

## Data Availability

Not applicable due to the type of manuscript.

## Abbreviations

ADPKD: Autosomal dominant polycystic kidney disease
CKD: Chronic kidney disease
eGFR: Estimated glomerular filtration rate
IBW: Ideal body weight
KA: Ketoacids
mTOR: Mammalian target of rapamycin
ND: Not on dialysis
PKD1: Polycystin 1
