# Supplementary figures and images for: Ketogenic diets in chronic kidney disease patients: a review for skeptics by skeptics
Source: J Nephrol. 2025 Apr 30;38(6):1541–56. doi: 10.1007/s40620-025-02285-7 (PMC12378136; doi:10.1007/s40620-025-02285-7)

**Table 1 suppl.** Some examples of commercial artificial products for ketogenic diets.


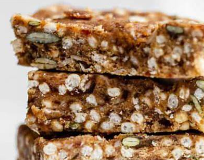

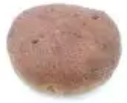

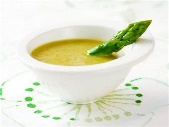

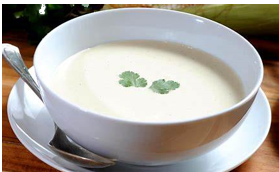

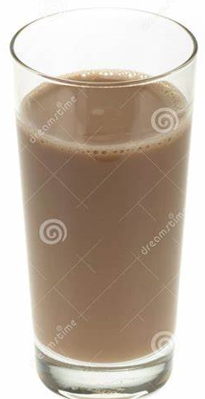

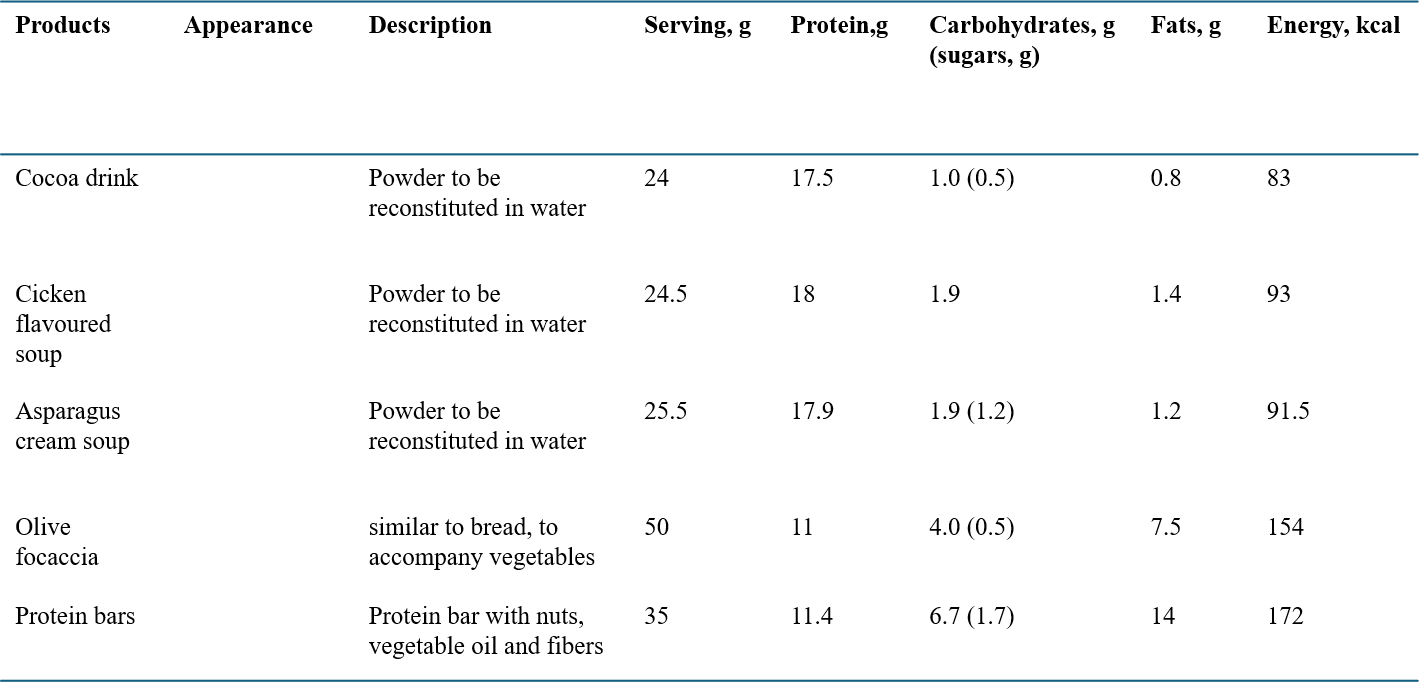

Supplement: Supplementary file 1 — Supplementary file1 (DOCX 312 KB) [file 40620_2025_2285_MOESM1_ESM.docx]
