# Supplementary material for: Ketogenic diets in chronic kidney disease patients: a review for skeptics by skeptics
Source: J Nephrol. 2025 Apr 30;38(6):1541–56. doi: 10.1007/s40620-025-02285-7 (PMC12378136; doi:10.1007/s40620-025-02285-7)
Supplement: Supplementary file 2 — Supplementary file2 (DOCX 19 KB) [file 40620_2025_2285_MOESM2_ESM.docx]

**Table 2 suppl.** Planning a very low-calorie ketogenic diet (VLCKD) in CKD and dialysis patients [43]

|  |  |  | |  |  |  |  |  |
| --- | --- | --- | --- | --- | --- | --- | --- | --- |
| CKD stage | | | Intensive phase | | Controlled phase | Maintenance phase | |  |
| 1. - 3a   Since stage 3a nephrologist is needed to discuss suitability.  Monitoring of renal function, electrolytes, acid-base and fluid status is recommended | | | 3 artificial meals (shakes)  + 2 cups low carbohydrate vegetables  + 1 teaspoon of oil  Liberal fluid intake  710 kcal  65 g protein  18 g fibre  31 mmol Na  92 mmol K  986 mg Ca  1171 mg P | | 2 artificial meals (shakes)  + 1 serving of dairy products or 300 ml of reduced fat milk  + 1 serving of fruit  Liberal fluid intake  947 kcal  72 g protein  19 g fibre  26 mmol Na  84 mmol K  1202 mg Ca  1193 mg P | 1 artificial meals (shakes)  + 1 light meals  + 2 servings of dairy products or 400 ml of reduced fat milk  + 2 servings of fruit  Liberal fluid intake  1308 kcal  84 g protein  31 g fibre  24 mmol Na  96 mmol K  1148 mg Ca  1314 mg P | |  |
|  | | |  | |  |  |  |  |
| 5 Dialyisis  Nephrologist is needed to discuss suitability case by case-.  Monitoring of renal function, electrolytes, acid-base and fluid status is recommended | | | 4 artificial meals (shakes)  + 1 cup low carbohydrate vegetables  + 1-2 teaspoon of oil  Fluid intake as per allowance  Or  3 artificial meals + a portion of protein powder or high protein food (defined by the dietitian)  912 kcal  85 g protein  22 g fibre  40 mmol Na  116 mmol K  1405 mg Ca  1531 mg P | | 2 artificial meals (shakes)  + 1 serving of dairy products or 150 ml of reduced fat milk  + 1 serving of fruit  Fluid intake as per allowance  1012 kcal  82 g protein  19 g fibre  27 mmol Na  87 mmol K  1207 mg Ca  1289 mg P | 1 artificial meal (shakes)  +2 light meals  + 1 servings of dairy products or 150 ml of reduced fat milk  + 2 servings of fruit  Fluid intake as per allowance  1439 kcal  104 g protein  31 g fibre  25 mmol Na  102 mmol K  1157 mg Ca  1506 mg P |  |  |
|  | | | Binders are recommended if phosphate level is outside the reference range, as well as in the case of hyperkalemia Monitor for constipation | | | | | |
