# Supplementary material for: Ketogenic diets in chronic kidney disease patients: a review for skeptics by skeptics
Source: J Nephrol. 2025 Apr 30;38(6):1541–56. doi: 10.1007/s40620-025-02285-7 (PMC12378136; doi:10.1007/s40620-025-02285-7)
Supplement: Supplementary file 3 — Supplementary file3 (DOCX 21 KB) [file 40620_2025_2285_MOESM3_ESM.docx]

**Table 3 supplementary.** A “toolkit” for the management of a very low-calorie ketogenic diet (VLCKD) in CKD patients.

| Side effects | Suggested intervention |
| --- | --- |
| Bloating, colitis, diarrhea | - Probiotics can be recommended to improve gut microbiota composition. To reduce symptoms product such as dimethicone may be suggested as well as fennel or lemon balm-based products, avoiding herbal products of uncertain composition due to possible interactions with drugs. Behavioral advice such as chewing well and eating slowly can be added |
| Constipation | -Hydration should be monitored. CKD patients are generally invited not to force hydration but in the case of a KD increased water intake is recommended. Attention should be paid in patients on dialysis.  - Increase fiber intake: Konjac pasta, rich in glucomannan, represents a source of fiber compensating for the low intake in KD.  A soluble fiber preparation without added sugar could be suggested or those who do not like Konjac pasta |
| Electrolytes alterations | -Monitoring sodium, potassium and magnesium is advisable in patients following KD. In CKD patients electrolytes monitoring is recommended as a regular routine and specific advises on food selection, cooking methods (namely the use of boiling) may be necessary.  -Use of potassium binders or changes in dialysate concentration (in the case of dialysis patients) must be evaluated on an individual basis.  -Electrolyte deficiencies are a possible occurrence: in this case supplementation must be evaluated on individual basis. |
| Gallstones | - Use of olive oil could prevent gallstones production. Use of ursodeoxycholic acid could be advisable |
| Halitosis | - Proper oral hygiene and use of mouthwashes (preferably hyaluronic acid based) |
| Headache (usually at the beginning of the diet) | - It is usually present at the beginning and it is temporary. - It is advisable to advise the patient not to take non-steroidal anti-inflammatory drugs |
| Hypoglycemia | - The restriction of carbohydrate intake need an adjustment of medications in patients with diabetes |
| Hypotension | -Blood pressure may decrease with weight loss.  -VLCKD may have a diuretic effect so adjustment in antihypertensive drugs may be required, diuretics in particular |
| Kidney stones | -Hydration should be monitored. CKD patients are generally invited not to force hydration but in the case of a KD increased water intake is recommended. Attention should be paid in patients on dialysis.  -the use of alkalizing agents could be advisable |
| Lipids profile alterations | - Monitoring is recommended to adjust lipid lowering drugs |
|  |  |
| Monitoring* |  |
| Parameter | **Frequency** |
| *Biochemistry* |  |
| Ketonuria (urine dipsticks) | - Once a week |
| Creatinine, blood urea, sodium, potassium, phosphorus, calcium, magnesium, serum bicarbonate, total cholesterol, HDL/LDL cholesterol, triglycerides, glycemia | - Every 4 weeks (unless otherwise necessary) |
| total protein, albumin, total uric acid, ALT. AST, gamma-GT, blood count, transferrin, iron, ferritin, 25-OH-vitamine D, C-reactive protein | - According to medical team follow up program |
| *Anthropometry and Body composition* | |
| Weight | - Once a week |
| Waist circumference | - Once a week |
| Bioimpedance analysis | - (If possible) once a month |

The table offers suggestions to counteract possible side effects, drugs adjustment and monitoring. As regards nutritional status evaluation, the table proposes a minimum evaluation. In CKD patients an accurate evaluation every 3-6 months (according to the CKD stage) is advisable.

*All the biochemical and body composition parameters must be evaluated

ALT: alanine aminotransferase, AST: aspartate aminotransferase,
